# Sweet Taste Dysgeusia in a Patient with Indapamide-Related Hyponatremia: Case Report and Review of the Literature

**DOI:** 10.7759/cureus.13079

**Published:** 2021-02-02

**Authors:** Pedro Gaspar, Filipe Bessa, Pedro Antunes Meireles, Inês Parreira, Catarina Mota

**Affiliations:** 1 Internal Medicine, Serviço de Medicina 2, Hospital de Santa Maria, Centro Hospitalar Universitário Lisboa Norte, Lisboa, PRT; 2 Internal Medicine, Faculdade de Medicina, Universidade de Lisboa, Lisboa, PRT; 3 Oncology, Serviço de Oncologia, Instituto Português de Oncologia de Lisboa Francisco Gentil, Lisboa, PRT; 4 Oncology, Serviço de Medicina 2, Hospital de Santa Maria, Centro Hospitalar Universitário Lisboa Norte, Lisboa, PRT

**Keywords:** dysgeusia, hypergeusia, sweet taste, paraneoplastic, hyponatremia, iatrogenic, indapamide

## Abstract

Sweet taste dysgeusia is a rare symptom where patients experience all food as having a sweet taste. While its cause is still unknown, it has been increasingly reported in the setting of lung cancer and syndrome of inappropriate secretion of antidiuretic hormone-related hyponatremia. In this case report, we present what we believe to be the first case of sweet taste dysgeusia in a non-cancer context. We will briefly review and summarize all published cases describing this symptom and also reflect upon the nature of this condition focusing on the role of serum sodium levels in sweet taste receptor modulation.

## Introduction

Dysgeusia is a distortion in taste perception, and, although uncommon, it is a well-recognized symptom of systemic conditions such as metabolic disorders (e.g., diabetes mellitus), ionic disturbances (e.g. zinc deficiency), and a secondary effect of some drugs (e.g., cisplatin) [1]. Such conditions tend to interfere with taste perception in a non-selective way, leading to the disturbance of more than one (usually all) types of taste [1]. Sweet taste dysgeusia is a rare type of partial dysgeusia where there is increased acuity for sweet perception only. This finding has been described in cases where hyponatremia developed in the setting of the syndrome of inappropriate secretion of antidiuretic hormone (SIADH) and lung cancer [2-9], raising the question of whether it could represent an alerting sign for clinicians for this paraneoplastic phenomenon. Even though this triad appears to be unique, we have previously presented a case of this specific taste distortion in a woman with hyponatremia secondary to indapamide use and in whom no cancer was found after approximately one year of follow-up [10]. Here we represent the same case after three years of follow-up in order to analyze it from a possible pathophysiological point of view.

## Case presentation

**Figure 1:**
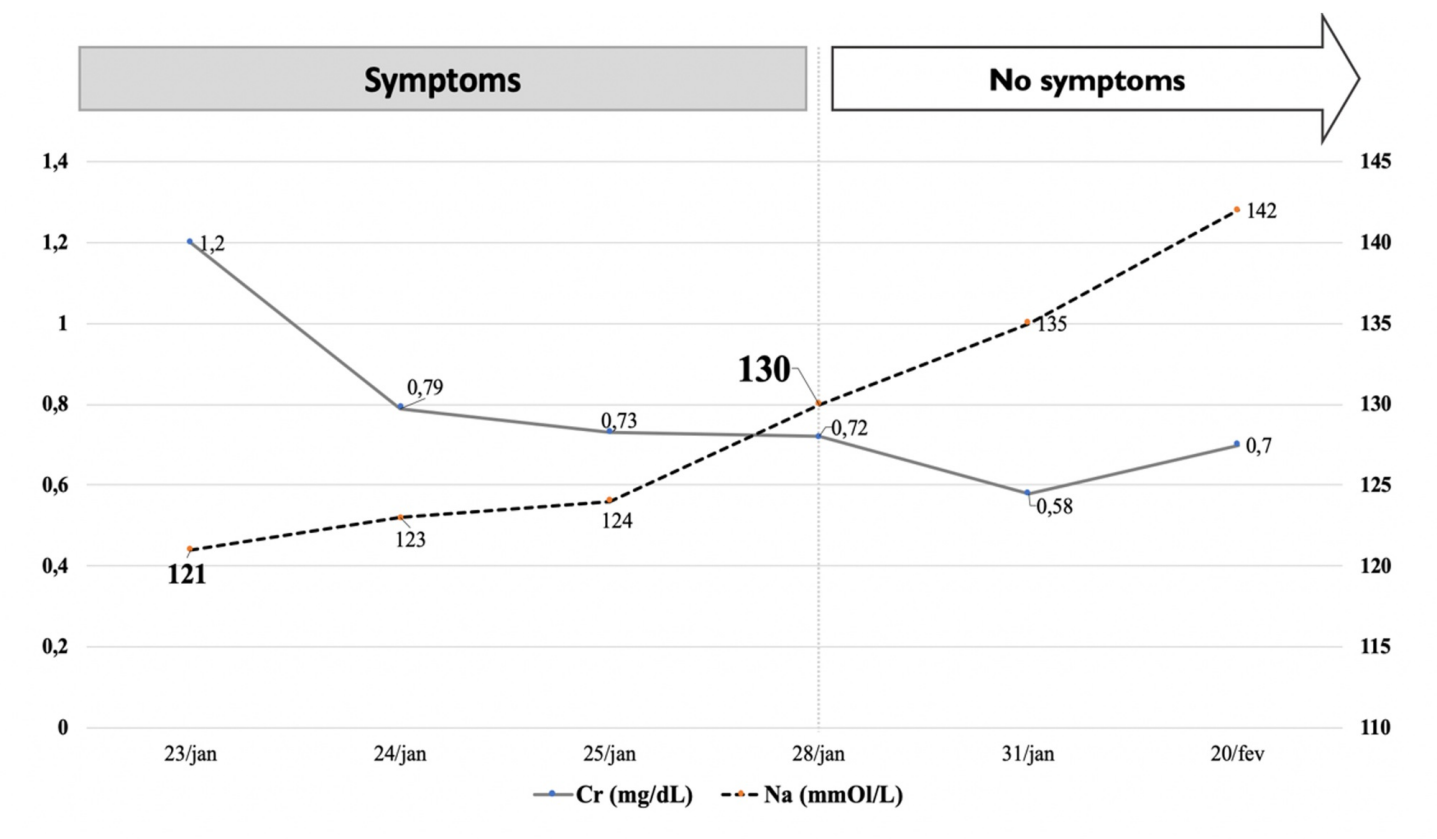
Evolution of symptoms in regard to renal function and sodium plasma concentration Abbreviations: Cr, serum creatinine levels; Na, sodium concentration levels

A 71-year-old woman presented to our medical ward with a three-week history of non-selective anorexia, asthenia, nausea, and weight loss (3 kg). She also complained of an unpleasant sweet taste in which all food was perceived as sweet. Her past medical history included essential hypertension and depression. She never smoked. Long-term medications included perindopril, amlodipine, and paroxetine. She was started on indapamide two weeks prior to the beginning of symptoms. On physical examination, she was slightly dehydrated, with no other relevant finding. Laboratory was as follow: occasional glucose of 171 mg/dL, serum urea of 37 mg/dL, serum creatinine of 1.2 mg/dL, sodium of 120 mmol/L, potassium of 2.2 mmol/L, zinc of 16.6 ug/dL, copper of 142 ug/dL, serum osmolality of 257 mOsmol/kg, and urinary osmolality of 133 mOsmol/kg. Chest X-ray did not show any finding, and the head and all body CT scans with intravenous contrast were unremarkable, with no signs of malignancy. Considering the diagnosis of iatrogenic hyponatremia and hypokalemia, indapamide and paroxetine were stopped, and electrolyte correction was started. Over the following days, the sweet taste sensation lessened as sodium concentration began to rise and finally disappeared when sodium concentration reached 130 mmol/L (Figure 1). In the following months, paroxetine was restarted. It has been three years of follow-up time. Until now, her serum sodium concentration remains at normal levels, and the sweet taste dysgeusia did not recur.

## Discussion

While the cause of the sweet taste dysgeusia remains largely unknown, sodium serum levels are likely to play a pivotal role in the pathophysiology of this intriguing symptom. The association between sweet taste dysgeusia and hyponatremia secondary to paraneoplastic SIADH was first brought to medical attention in 1995 when Panayiotou et al. [2] described three patients who were diagnosed with small cell carcinoma of the lung after the onset of sweet taste dysgeusia related to hyponatremia. Seven other patients have been reported since then and their clinical similarities are a striking feature [3-9].

Table 1 sums all 10 cases found in the literature.

**Table 1:** Characteristics from case series and case reports on cancer-related sweet taste dysgeusia Abbreviations: F, female; M, male; Na, sodium plasma concentration; SIADH, syndrome of inappropriate secretion of antidiuretic hormone

Author, year	Patient (sex/age in years)	Clinical picture	Duration of symptoms (weeks)	Na (mmol/L)	Diagnosis
Panayiotou et al. [2]	M/63	Sweet dysgeusia	3	114	SIADH/small cell lung carcinoma
	F/63	Sweet dysgeusia, nausea, and weight loss	4	116	SIADH/small cell lung carcinoma
	M/60	Sweet dysgeusia, pulmonary mass	8	116	SIADH/small cell lung carcinoma
Croghan and Salik [3]	F/59	Sweet dysgeusia, dyspnea, nausea, weight loss	12	118	SIADH/small cell lung carcinoma (with hepatic metastasis)
Karthik et al. [4]	F/69	Sweet dysgeusia	unknown	122	SIADH/lung adenocarcinoma
Nakazato et al. [5]	F/56	Sweet dysgeusia, nausea, and weight loss	7	113	SIADH/lung neuroendocrine carcinoma
Ellison and Berl [6]	M/62	Sweet dysgeusia	unknown	122	SIADH/small cell lung carcinoma
Singh et al. [7]	F/60	Sweet dysgeusia, loss of consciousness, nausea, and weight loss	10 days	111	SIADH/small cell lung carcinoma (with non-specified metastasis)
Eshuis et al. [8]	F/70	Sweet dysgeusia, nausea, and weight loss	4	125	SIADH/small cell lung carcinoma (with hepatic metastasis)
Schuermann et al. [9]	F/60	Sweet dysgeusia	8	110	SIADH/small cell lung carcinoma (with non-specified metastasis)

All patients described the sweet dysgeusia as an unpleasant sweet taste where most foods were perceived as sweet, and, most importantly, this complaint was the reason that led them to seek for a medical observation [2-9]. Small cell carcinoma was the most frequent histological type, whereas neuroendocrine large cell carcinoma and adenocarcinoma were reported in two patients only [4,5]. All patients had hyponatremia, which was clearly linked with the severity of the symptoms. All patients had their symptoms lessened upon hyponatremia correction [2-9], and, more importantly, some of them recurred during hyponatremia reappearance [4,5,7].

Pure sweet taste loss is described in anti-acetylcholine receptor antibody (AChRAb)-myasthenia gravis (MG) patients [11,12]. The progressive loss of sweet taste appears to follow the disease activity as dysgeusia evolved in parallel with MG composite score [12] and AChRAb titers [11]. Importantly, this sweet hypogeusia always coexists with thymoma in MG, suggesting a paraneoplastic autoimmune phenomenon targeting the G-protein coupled sweet receptor [12]. Therefore, it is reasonable to think that malignant neoplasms may produce unknown taste modifying substances that might induce structural changes in the sweet taste-receptor membrane [5].

Serotonin is an important neurotransmitter in taste reception signaling [13,14]. In healthy volunteers, the serotonin selective receptor inhibitor (SSRI) paroxetine significantly increases sweet taste sensitivity (p < 0.001) [15], and sertraline treated successfully an elderly patient with mild depression who described every food as having no taste [16].

The pathophysiology of this special type of dysgeusia is probably multifactorial since none of the aforementioned reasons explains it by itself. Hyponatremia is the most common electrolyte disturbance in hospitalized patients [17], and, yet, most patients will not develop taste perception abnormalities. Similarly, even though lung cancer is the most commonly diagnosed cancer worldwide [18], taste disturbances are uncommon prior to treatment [19].

In the present case, our patient described the same sweet taste dysgeusia as the cases mentioned before, but no malignant neoplasm was found, particularly lung cancer. In contrast to the reported cases where SIADH was the cause of hyponatremia [2-9], in our case, the variation in sodium concentration with indapamide introduction and withdrawal, the presence of hypokalemia, and the improvement of serum sodium concentration without water restriction favor the hypothesis of hyponatremia secondary to diuretic use. SSRI drugs are a well-known cause of hyponatremia, particularly in the elderly [20]. Our patient was on paroxetine for over 15 years. Despite this, she had never had this symptom before. We stopped paroxetine as a first attempt to assess our patient’s hyponatremia. Later on, paroxetine was restarted, and, until now, her serum sodium levels remain at normal range, and the sweet taste dysgeusia did not recur.

Overall, it appears that in order to develop sweet dysgeusia, one must have a combination of factors that potentiate the action of one another and would ultimately modulate the sweet taste receptor. Hyponatremia is the only common characteristic in the aforementioned cancer-related cases and ours. It is thus possible that our patient developed a sweet taste dysgeusia due to indapamide-related hyponatremia in the context of an already modulated sweet taste receptor environment by paroxetine. We could, herein, hypothesize that serum sodium concentrations not only have a modulating effect on the sweet taste receptor but also are the main driver of this particular sweet taste disturbance.

## Conclusions

We present what we believe to be the first case report of sweet taste dysgeusia in a non-cancer context. In light of the literature, our case further underlines the particular role that sodium plasma levels have on the modulation of the sweet taste receptor. Even though we did not find any malignant neoplasm, we strongly consider that one should not be discouraged to pursue a cancer workout in the setting of this rare symptom.
